# fqfa: A pure Python package for genomic sequence files

**DOI:** 10.21105/joss.02076

**Published:** 2020-03-01

**Authors:** Alan F. Rubin

**Affiliations:** 1 The Walter and Eliza Hall Institute of Medical Research; 2 The University of Melbourne

## Summary

Modern bioinformatics requires the use of many field-specific file formats. Two of the most prevalent formats for representing biological sequences are FASTA (Pearson & Lipman, 1988) and FASTQ (Cock, Fields, Goto, Heuer, & Rice, 2010). While multiple feature-rich Python bioinformatics libraries exist that can process biological sequence files (Cock et al., 2009; scikit-bio Development Team, 2013), they require complex compiled dependencies that may limit their use in non-Unix environments. Other FASTA or FASTQ specific Python libraries (Du, 2019; Hunt, 2013; Pedersen, 2010; Shirley, Ma, Pedersen, & Wheelan, 2015) are outdated, require runtime dependencies, or make heavy use of C extensions that prioritize speed over readability and portability.

fqfa is a pure Python package that aims to fill the needs of bioinformatics and computational biology researchers who want a simple and efficient solution for working with files in FASTA and FASTQ formats. It has no dependencies outside of the Python standard library (with the exception of backported dataclasses (Smith, 2017) for Python 3.6 users) and makes use of newer language features such as type hinting and f-strings to improve readability. These implementation details make fqfa highly suitable for use in notebooks and projects that have simple requirements, with underlying code that is easy for novice bioinformaticians and students to understand and explore.

Although fqfa is written in pure Python, its performance is comparable to modules using C extensions like pyfastx (Du, 2019) for tasks such as processing a FASTQ file sequentially and collecting or filtering on quality statistics from the high-throughput sequencing reads. Detailed benchmarking results and usage examples comparing fqfa and pyfastx (Du, 2019) are available as part of the fqfa documentation in static format as well as in Jupyter notebooks (Kluyver et al., 2016).

fqfa is released under the BSD 3-Clause License and is available from GitHub and PyPI.
